# On a fractional order calculus model in diffusion weighted breast imaging to differentiate between malignant and benign breast lesions detected on X-ray screening mammography

**DOI:** 10.1371/journal.pone.0176077

**Published:** 2017-04-28

**Authors:** Sebastian Bickelhaupt, Franziska Steudle, Daniel Paech, Anna Mlynarska, Tristan Anselm Kuder, Wolfgang Lederer, Heidi Daniel, Martin Freitag, Stefan Delorme, Heinz-Peter Schlemmer, Frederik Bernd Laun

**Affiliations:** 1 German Cancer Research Center (dkfz), Department of Radiology, Heidelberg, Im Neuenheimer Feld 280, Heidelberg, Germany; 2 German Cancer Research Center (dkfz), Medical Physics in Radiology, Heidelberg, Im Neuenheimer Feld 280, Heidelberg, Germany; 3 Radiological Clinic at the ATOS Clinic Heidelberg, Heidelberg, Bismarckplatz 9–15, Heidelberg, Germany; 4 Radiology Center Mannheim (RZM), Mannheim, Rosengartenplatz 7, Mannheim, Germany; 5 University Hospital Erlangen, Department of Radiology, Maximiliansplatz 3, Erlangen, Germany; Nanjing University, CHINA

## Abstract

**Objective:**

To evaluate a fractional order calculus (FROC) model in diffusion weighted imaging to differentiate between malignant and benign breast lesions in breast cancer screening work-up using recently introduced parameters (*β*_FROC_, *D*_FROC_ and *μ*_FROC_).

**Materials and methods:**

This retrospective analysis within a prospective IRB-approved study included 51 participants (mean 58.4 years) after written informed consent. All patients had suspicious screening mammograms and indication for biopsy. Prior to biopsy, full diagnostic contrast-enhanced MRI examination was acquired including diffusion-weighted-imaging (DWI, b = 0,100,750,1500 s/mm^2^). Conventional apparent diffusion coefficient *D*_app_ and FROC parameters (*β*_FROC_, *D*_FROC_ and *μ*_FROC_) as suggested further indicators of diffusivity components were measured in benign and malignant lesions. Receiver operating characteristics (ROC) were calculated to evaluate the diagnostic performance of the parameters.

**Results:**

29/51 patients histopathologically revealed malignant lesions. The analysis revealed an AUC for *D*_app_ of 0.89 (95% CI 0.80–0.98). For FROC derived parameters, AUC was 0.75 (0.60–0.89) for *D*_FROC_, 0.59 (0.43–0.75) for *β*_FROC_ and 0.59 (0.42–0.77) for *μ*_FROC_. Comparison of the AUC curves revealed a significantly higher AUC of *D*_app_ compared to the FROC parameters *D*_FROC_ (p = 0.009), *β*_FROC_ (p = 0.003) and *μ*_FROC_ (p = 0.001).

**Conclusion:**

In contrast to recent description in brain tumors, the apparent diffusion coefficient *D*_app_ showed a significantly higher AUC than the recently proposed FROC parameters *β*_FROC_, *D*_FROC_ and *μ*_FROC_ for differentiating between malignant and benign breast lesions. This might be related to the intrinsic high heterogeneity within breast tissue or to the lower maximal b-value used in our study.

## Introduction

Breast cancer screening programs have been established in many countries in order to reduce the burden of breast cancer in the female population [1–3]. While substantial evidence has been provided regarding the benefit of these programs using X-ray mammography as the screening tool, the high rate of false positive findings triggering unnecessary invasive biopsies and potential overdiagnosis have repeatedly been criticized [2, 4].

Current reports from organized, quality-assured breast cancer screening programs report a substantial false-positive rate of about 50% in invasive biopsies [5]. Alternative or additive imaging modalities to address this issue have therefore been introduced and are currently being investigated regarding the benefit in a screening environment [6, 7]. Amongst them, magnetic resonance imaging (MRI) has been a promising method, since abbreviated protocols might allow the use in a screening environment and provide a high sensitivity for detecting malignancy exceeding that of X-ray mammography [7].

In addition to contrast enhanced protocols, abbreviated unenhanced protocols omitting contrast agent administration have been subject of different studies with promising results regarding the differentiation between malignant and benign breast lesions [8–11]. Besides shortening examination times, another aspect of unenhanced, abbreviated breast MRI protocols represent recent reports on gadolinium deposition in the human brain after repetitive intravenous application with unclear clinical relevance [12, 13].

One sequence of increasing interest in breast MRI protocols is the diffusion weighted imaging (DWI) sequence [14–16], which is thought to visualize the free Brownian water motion [15, 17–19]. Highly packed cell conglomerates restrict water diffusion thus leading to signal intensity changes on diffusion weighted images.

DWI images are commonly used to calculate apparent diffusion coefficient (*D*_app_) maps. Those maps allow quantifying diffusion restriction, but the clinical acceptance is limited related to the overlap between malignant and benign lesions. Other diffusion models have therefore been suggested aiming to improve the differentiation between tissue structures, e.g. in order to represent the tissue heterogeneity. Increased tissue heterogeneity can be assumed in many malignant entities and might be as well of interest in breast imaging. Malignant cell conglomerates might demonstrate an increased heterogeneity as compared to benign lesions since breast cancer cells have been described as morphologically highly variable [20]. One of the diffusion models aiming to produce values that are thought to be related to tissue heterogeneity is the fractional order calculus model (FROC) [21–25]. Using the FROC measures *β*_FROC_, *D*_FROC_ and *μ*_FROC_ has recently been proposed to contribute to the differentiation of brain tumors exceeding a conventionally calculated apparent diffusion coefficient *D*_app_ [21].

Therefore this study aimed at investigating whether the previously proposed new FROC derived parameters *β*_FROC_, *D*_FROC_ and *μ*_FROC_ as a measure of tissue heterogeneity might be also of diagnostic value in breast imaging in order to differentiate between malignant and benign lesions using unenhanced breast MRI as compared to the apparent diffusion coefficient (*D*_app_).

## Materials and methods

### Patients

This study was conducted as a retrospective subgroup analysis of an ongoing larger prospective multicenter study on DWI in breast imaging with written informed consent and institutional and governmental review board approval. Preliminary results of patients in one study site that received acquisition of a vendor specific DWI sequence (Diffusion Weighted Imaging With Background Suppression, DWIBS) have been published regarding the usability of different visual radiologists´ reading strategies of an abbreviated breast MRI protocol for breast cancer screening work-up and in regards to so called radiomics analyses of the DWI ()[9, 26–28]. Mathematical analysis of the FROC derived parameters *β*_FROC_, *D*_FROC_ and *μ*_FROC_ as evaluated in this study here have not been part of these previously published analyses and the patients described originate from a different study site. Since all patients belong to one multicenter study concept, however, data, principles and background of the methods described here may partially overlap to the previous reports [9, 26–28].

51 patients (mean age 58 years; SD ± 6.2) were analyzed out of the recruitment period between May 2015 and February 2016. Inclusion criteria were: female participants of the national breast cancer screening program with a BIRADS 4/5 lesion in the primary screening X-ray mammogram; a regular subsequent screening work-up process that included clinical, ultrasonographic (US) and, if necessary, repeat mammography examinations; final indication for biopsy in concordance to the screening guidelines and the Breast Imaging Reporting and Data System (BI-RADS) category 4 or 5.

### MR imaging

Patients participating in the study received a MR examination prior biopsy as previously described [26]. The MR examination consisted of a full diagnostic protocol including unenhanced morphologic sequences (T1-weighted, T2-weighted), contrast enhanced sequences (0.1 mmol Gadobenate Dimeglumine (Multihance; Bracco, Mailand, Italy) per kilogram of body weight) and diffusion weighted sequences with details described in Table 1. MR imaging examinations were performed using a 1.5-T MR imaging unit (Aera, Siemens, Erlangen, Germany) with a dedicated 18-channel breast coil. Participants were placed in prone position with the breasts not compressed but softly fixed using foamed material.

**Table 1:** Sequence parameters.

	Slice thickness (mm)	FOV (mm x mm)	Voxel Size	Orientation	TE (ms)	TR (ms)	b-value s/mm^2^	Phase enconding direction	Additional feature
**Localizer**									
**T1w TSE**	3	384x384	1x1.3	Coronal	8	886	-	RL	Grappa x2
**T2w fs**	3	263x350	0.9x0.6	Transverse	82	8490	-	RL	“TIRM”
**T2w TSE**	3	263x350	0.9x0.6	Transverse	120	6710	-	RL	Grappa x2
**DWI**	3	480x240	2.5 x 2.5	Transverse	80	11700	b_1_ = 0, b_2_ = 100, b_3_ = 750, b_4_ = 1500	AP	SPAIR, Parallel imaging: Grappa x2, EPI-factor 96; Number of averages: 2
**T1w DCE**	1	350x263	1.0x0.6	Transverse	4.6	11	-	RL	6 dynamic pre-/post-contrast series, each 1.23 min; Grappa x2
**T1w TSE**	2	156/343	1.2/0.8	Transverse	4.6	30	-	AP	3D

Abbreviations: TSE = Turbo Spin Echo, FOV = Field of View, TE = Echo Time, TR = Repetition Time, RL = Left-Right, AP = Anterior-Posterior, SPAIR = Spectral Attenuated Inversion Recovery; TIRM = Turbo Inversion Recovery Magnitude, DCE = dynamic contrast-enhanced; GRAPPA = generalized autocalibrating partially parallel acquisition, mm = millimeter.

The DWI sequence was run with multiple b-values to allow for further fitting strategies. The upper limit of the b-values was chosen in order to provide a high specificity for displaying suspicious lesions while preserving sufficient suppression of other tissues. The DWI sequence was acquired prior contrast agent administration to avoid a potential influence of gadolinium on the DWI signal. The following parameters were applied: Echo Time (ms) 80; Repetition Time (ms) 1170; b-values 0, 100, 750, 1500 s/mm^2^; Spectral attenuated Inversion Recovery (SPAIR) fat suppression; Parallel Imaging, EPI-factor 96; separation between two diffusion gradient lobes 35.1 ms; duration of each diffusion gradient 14.1 ms; field of view 480 x 240 mm^2^; slice thickness 3 mm; imaging time 6:44 minutes; 50 slices.

### Image analysis

For imaging analysis, a previously published methodology for FROC analyses in brain tumors for the analysis of breast lesions was adapted [21, 22].

Regions of interest (ROIs) were drawn at the inner border of the lesion that was indicated for biopsy by using the images acquired with *b* = 1500 s/mm^2^. ROIs were placed slice by slice for the respective lesion creating a 3-dimensional volume for each lesion. Lesions were correlated to the X-ray mammogram using a visual correlation by two radiologists (*blinded*, <1 year experience, *blinded*, >5 years experience) in consensus, since correlation by means of invasive markers (clips) was not available.

The FROC model was used to calculate the voxel intensity within a diffusion weighted image as given by the equation

$$S=S_{0}\exp\;\left(-D_{FROC}\mu_{FROC}^{2(\beta_{FROC}-1)}{\left(\gamma G\delta \right)}^{2\beta_{FROC}}(\Delta -\frac{2\beta -1}{2\beta +1}\delta )\right)$$ (1)

In this equation, *S*_0_ is the signal intensity as given without diffusion weighting, *D*_FROC_ is the FROC diffusion coefficient, *β*_FROC_ is the fractional order derivative in space, *G* is the diffusion gradient amplitude, *δ* is the diffusion gradient pulse width, Δ is the gradient lobe separation [21–23].

The apparent diffusion coefficient *D*_app_ was calculated using a monoexponential fit using images acquired with *b* = 0 s/mm^2^, *b* = 100 s/mm^2^ and *b* = 750 s/mm. The fitting of the FROC parameters was performed in adaption to previous studies [21, 22] in a voxel by voxel manner using the Levenberg-Marquardt non-linear fitting method [21]. The initial *D*_FROC_ value was obtained from images at b-values ≤750 s/mm^2^ using the monoexponential fit. The initial *β*_FROC_ value was set to 0.5. Using these initial values for *D*_FROC_ and *β*_FROC_, the initial *μ*_FROC_ value was determined by fitting Eq 1 with *μ*_FROC_ being the only free variable. Afterwards, FROC parameters *β*_FROC_, *D*_FROC_ and *μ*_FROC_ were calculated with the Levenberg-Marquardt algorithm using the b-values 100, 750, and 1500 s/mm^2^. *S*_0_ was not fitted but set to the signal value at *b* = 0. This fitting approach is reproducible, i.e. performing this procedure twice yields identical results. However, *D*_FROC_ and *μ*_FROC_ are strongly coupled in Eq 1, so that the obtained values of these parameters were found to depend on their initial values. This limitation is described in more detail in the Supplemental Information.

The choice of b-values in this study allowed for an additional evaluation using the IVIM model [29]. IVIM parameters were calculated using using all b-values fitting with the Levenberg-Marquardt algorithm:

$$S=S_{0}[f_{IVIM}exp(-bD_{IVIM}^{*})+(1-f_{IVIM})\exp\left(-bD_{IVIM}\right)],$$

where $D_{IVIM}^{*}$ is the pseudo diffusion coefficient and *f*_IVIM_ is the perfusion fraction. $D_{IVIM}^{*}$ was fitted, but not used for further analysis due to the observed large fit instability.

Lesion size was measured using T2-weighted images with the maximal lesion diameter being measured in axial orientation by one reader.

To analyze a potential influence of lesion size, lesions were separated into two groups (below and above 10 mm maximal in plane diameter) thus defining a clinical threshold. The quantitative FROC model analysis was performed individually for these two groups.

All image processing was performed using software code developed with Matlab (MathWorks, Natick, Massachusetts).

### Histopathology

All patients participating this study underwent core-needle biopsy of the suspicious lesion as indicated in the screening clarification process. Histopathological analyses served as the standard of reference regarding the classification in benign or malignant lesions.

### Statistical analysis

For each patient, the values of the parameters (*D*_app_, *β*_FROC_, *D*_FROC_ and *μ*_FROC_) and of the IVIM parameters were calculated for each voxel within the Volume of Interest. For both, the malignant and the benign lesions, mean and 25% - 75% percentile were calculated and compared using Mann-Whitney U test after normality testing with Shapiro-Wilk test failed.

Receiving operating characteristics (ROC) were calculated in order to evaluate the area under the ROC curve (AUC) and to assess the performance of each individual parameter.

Significant differences were considered for a p-value <0.05. Statistics were calculated using SigmaPlot (Systat Software; Erkrath, Germany).

## Results

### Cancer burden and histopathological characteristics within the study population

Of the 51 patients in the study, 29 (58%) had a malignancy. Of the malignant lesions, most lesions were described as invasive ductal carcinoma (IDC; n = 26; 89.6%), two lesions were invasive lobular carcinoma (ILC; 6.8%) and one lesion was a ductal carcinoma in situ (DCIS; 3.4%). Of the benign lesions, six lesions were fibroadenoma (27.3%), five lesions were revealed as fibrosis (22.7%), three lesions were papilloma (13.6%), two lesions each were usual ductal hyperplasia (UDH, 9.1%), compacted breast tissue (9.1%) and fibrocystic mastopathy (9.1%), and one lesion each was fat tissue (4.5%) and granulomatous lymphadenitis (4.5%). (see Table 2 for details).

**Table 2:** Type and distribution of lesions within the study population.

		Number	Percentage
**Benign**	Granulomatous lymphadenitis	n = 1	4.%
	Compacted breast tissue	n = 2	9.1%
***(n = 22)***	Ductal hyperplasia	n = 2	9.1%
***(52%)***	Fibroadenoma	n = 6	27.3%
	Fibrosis	n = 5	22.7%
	Fibrocystic mastopathia	n = 2	9.1%
	Papilloma	n = 3	13.6%
	Fat tissue	n = 1	4.5%
**Malignant**			
***n = 29***	Invasive ductal carcinoma (IDC)*	n = 26	89.6%
***(58%)***	carcinoma in situ (DCIS)	n = 1	3.4%
	Invasive lobular carcinoma	n = 2	6.8%
***Lesion Size***		**Mean**	**SD**
	**Benign**	11.54 mm	4.03 mm (range 4.9–21.7)
	**Malignant**	12.56 mm	5.17 mm (range 6.3–21.3)

### Lesion size

Mean lesion size for benign breast lesions was 11.54 mm (SD ± 5.16 mm) with a range from 4.9–21.7 mm. Malignant lesion had a mean lesion size of 12.54 mm (SD ± 4.03 mm) with a range of 6.3–21.3 mm without significant difference to the benign lesion (p = 0.18).

### Diagnostic performance of apparent diffusion coefficient *D*_app_, fractional order calculus (FROC) derived parameters and intravoxel incoherent motion (IVIM) derived parameter

**Fig 1:**
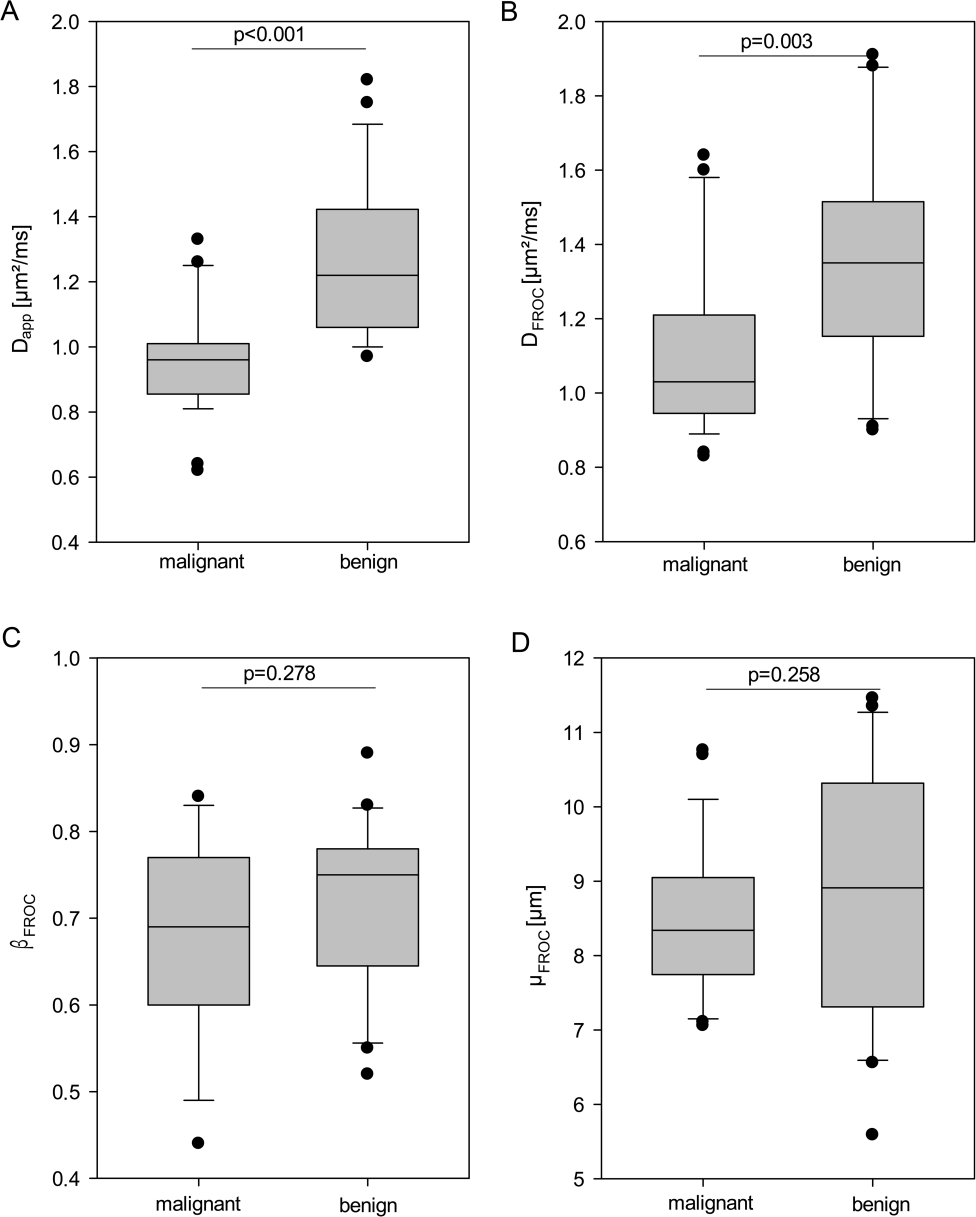
Boxplots. Boxplots of the mean values for conventional apparent diffusion coefficient D_app_ (A) and fractional order calculus (FROC) model derived parameters D_FROC_ (B), β_FROC_ (C) and μ_FROC_ (D) for benign and malignant lesions. Vertical bars mark the range of the data excepting outliers, dots mark outliers, box marks 25^th^– 75^th^ percentile, horizontal bar marks median.

*D*_app_ was significantly decreased (p<0.001) in malignant lesions (median 0.97 μm^2^/ms; 25% - 75% percentile 0.87–1.01 μm^2^/ms) compared to benign lesions (1.20 μm^2^/ms; 1.06–1.47 μm^2^/ms) (Fig 1). Analysis of the Area Under the Curve (AUC) revealed an AUC of 0.89 (95% CI 0.79–0.974) for *D*_app_.

Fractional Order Calculus (FROC) derived parameters *β*_FROC_ and *μ*_FROC_ revealed no statistically significant difference between malignant and benign lesions (for *β*_FROC_ p = 0.278, for *μ*_FROC_ p = 0.258) (Fig 1). In detail, *β*_FROC_ revealed a median of 0.69 (25%–75% percentile 0.60–0.77) for malignant and 0.75 (25%–75% percentile 0.64–0.78) for benign lesions. *μ*_FROC_ resulted in a mean of 8.34 μm (25%–75% percentile 7.74–9.05) for malignant and 8.91 μm (25%–75% percentile 7.30–10.30) for benign lesions. *D*_FROC_ revealed a significant difference between malignant and benign lesions with a *D*_FROC_ of 1.03 μm^2^/ms (25%–75% percentile 0.94–1.2) for malignant and 1.35 μm^2^/ms (25%–75% 1.14–1.51) for benign lesions (p = 0.003).

The AUC analysis revealed an AUC for *D*_app_ of 0.89 (95% CI 0.80–0.98) and for *D*_FROC_ of 0.75 (0.60–0.89). FROC parameter *β*_FROC_ showed an AUC of 0.59 (0.43–0.75) and *μ*_FROC_ revealed an AUC of 0.59 (0.42–0.77).

**Fig 2:**
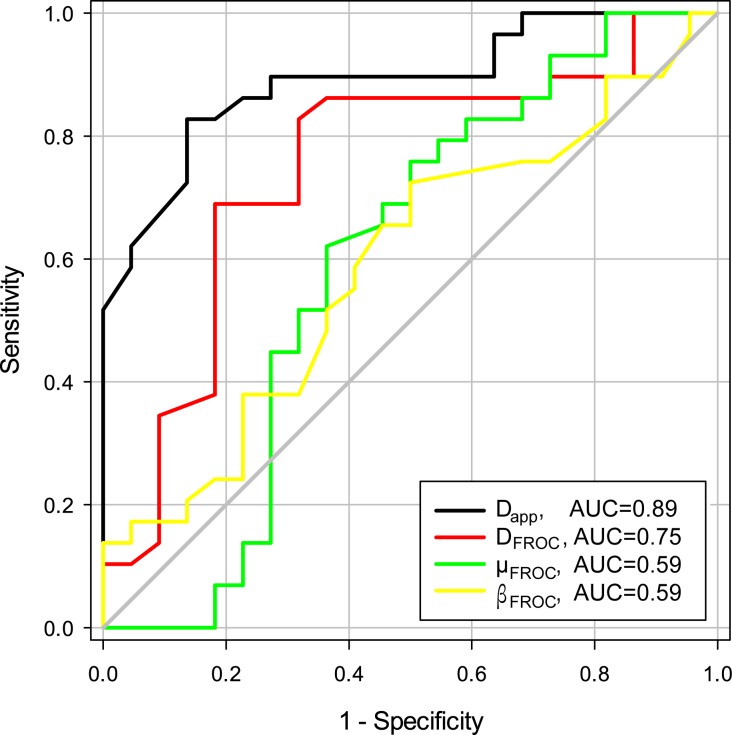
Receiving operator characteristics (ROC) curves. ROCs visualizing the diagnostic accuracy of the diffusion coefficient (D) and FROC derived parameters β_FROC_, μ_FROC_, and D_FROC_.

**Fig 3:**
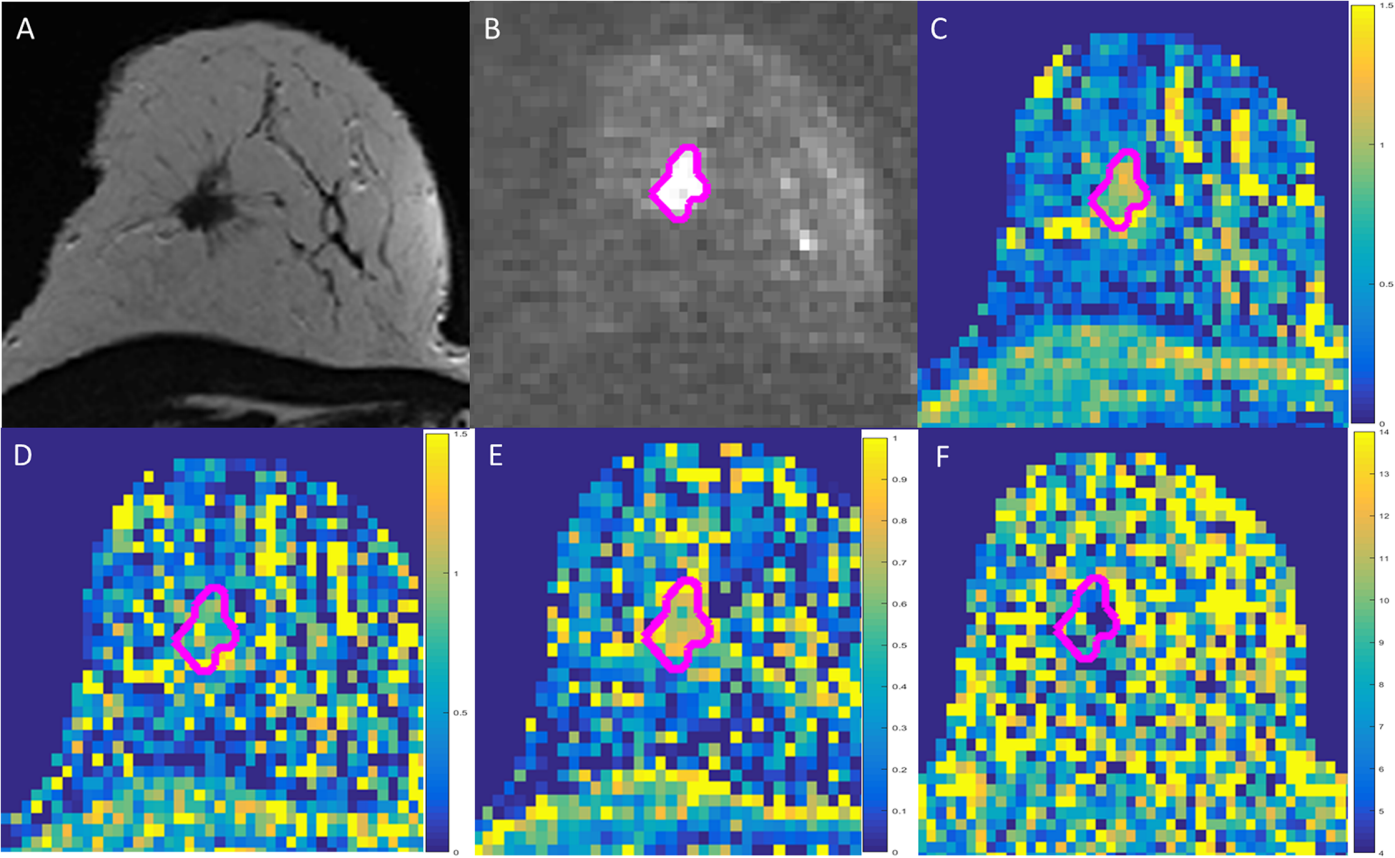
Example of a breast cancer screening participant (66 years). Lesion with segmentation demonstrated as pink line. A) T2-weighted morphological sequence; B) diffusion weighted imaging (DWI, b = 1500 s/mm^2^); C) diffusion coefficient map D_app_ (scale given in μm^2^/ms); D) FROC diffusion coefficient map (scale given in μm^2^/ms); E) β_FROC_-map demonstrating a more heterogeneous signal; F) μ_FROC_-map demonstrating as well a relatively heterogeneous signal within the entire breast. Histopathology: invasive ductal carcinoma (IDC).

Individual comparison of the AUC curves revealed a significantly higher AUC of *D*_app_ compared to the FROC parameters *D*_FROC_ (area difference 0.14; p = 0.009), *β*_FROC_ (area difference 0.29; p = 0.003) and *μ*_FROC_ (area difference 0.29; p = 0.001) (Fig 2, image examples are shown in Fig 3).

The IVIM parameter *D*_IVIM_ revealed an AUC of 0.91 (95% CI 0.82–0.99) and for *f*_IVIM_ and AUC of 0.54 (0.37–0.69) was found.

Evaluation of the size dependence of the AUC curves for the different parameters revealed a tendency for increasing AUC with larger sizes for *D*_app_, *D*_FROC_ and *μ*_FROC_. Dividing the lesions into groups larger or smaller than 10 mm revealed for lesions >10 mm an AUC of *D*_app_ of 0.92 (95% CI 0.82–1.0) and for *D*_FROC_ of 0.81 (0.63–0.99). FROC parameter *β*_FROC_ showed an AUC of 0.59 (0.37–0.81) and *μ*_FROC_ revealed an AUC of 0.60 (0.35–0.86). For lesions smaller than 10 mm the AUC of *D*_app_ decreased to 0.86 (95% CI 0.61–1.0) and for *D*_FROC_ an AUC of 0.64 (0.32–0.96). FROC parameter *β*_FROC_ showed an AUC of 0.66 (0.38–0.93) and *μ*_FROC_ had an AUC of 0.52 (0.23–0.80).

A summary of the results is shown in Table 3.

**Table 3:** Area, 95% confidence interval and asymptotic significance of ROC curves.

	FROC parameters			apparent diffusion coefficient
	*D*_FROC_	*β*_FROC_	*μ*_FROC_	*D*_app_
**Area**	0.75	0.59	0.59	0.89
**95% confidence interval**	0.60–0.89	0.43–0.75	0.42–0.77	0.80–0.98
**p value**	p = 0.002	p = 0.27	p = 0.25	p<0.0001

## Discussion

Here we demonstrate that using the conventional apparent diffusion coefficient *D*_app_ in diffusion weighted breast imaging provides significantly different values between malignant and benign breast lesions. In contrast, out of the recently proposed FROC diffusion parameters *β*_FROC_, *μ*_FROC_ and *D*_FROC_, only the latter parameter revealed significant differences between malignant and benign lesions. The other parameters derived from the fractional order calculus model did not reliably differentiate between malignant and benign breast lesions detected in X-ray mammography. Comparing the different parameters, *D*_app_ resulted in a higher diagnostic accuracy in terms of AUC for predicting malignancy.

This is in concordance to existing literature and emphasizes the robust nature of the *D*_app_ for lesion characterization in the breast. Different studies have provided evidence that *D*_app_ values might help distinguish between malignant and benign breast lesions [14, 17–19]. However, in a similar manner as imaging features, *D*_app_ values show some overlap between malignant and benign lesions providing evidence for the necessity of further research. Common approaches to calculate the *D*_app_ values rely on monoexponential or biexponential models of diffusion within the imaged area/voxel [30–32]. However, potential intravoxel heterogeneity of the diffusion process might be underrepresented using these methods. A recently proposed new approach to estimate diffusions parameters suggested to take into account potential further diffusion derived parameters is the fractional order calculus model (FROC) [21–23]. Amongst those FROC parameters, *β*_FROC_ is a parameter recently described differentiating high- from low-grade glioblastomas by Sui et al. and described as mathematically equivalent to a heterogeneity measure described by Bennett et al. [21, 33]. Further parameters calculated with the FROC model are *μ*_FROC_ and *D*_FROC_. *μ*_FROC_ had previously been described as inferior in the grading of glioblastomas [21, 22].

The potential interdependence between *μ*_FROC_ and *D*_FROC_, which we consider as a limitation (see Supplemental Information: S1 Text: On the coupling of *D*_FROC_ and *μ*_FROC_; S1 Fig), might contribute to this fact as well as the fact that our study used a spectrum of b-values in the lower spectrum of previously described data [21–23] (see Supplemental Information: S2 Text: Choice of b-values;; S2 Fig). In addition, relevant general limitations of *μ*_FROC_ were already described by Zhou et al. since the calculation of *μ*_FROC_ becomes increasingly unstable regarding certain variations of *β*_FROC_ [21, 22].

In contrast to a previous study in brain tumors [21], FROC parameters did not perform as well as conventional apparent diffusion coefficient *D*_app_ in characterizing breast lesions. This might be related to the fact that the FROC parameter *β*_FROC_ is thought to be correlated to structural complexity [23]. Thus, there might be underlying biophysiological properties influencing the applicability of this complex FROC model in certain tissue components [21–23]. FROC parameters thus might be of dedicated value in differentiating changes within a more homogenous tissue such as the brain [21]. The breast tissue, especially in cases of fibrocystic changes and mastopathic tissue, already presents itself in a high structural complexity without interpreting the pure presence of structural complexity and microstructural heterogeneity as a dedicated index of malignancy. Within such a complex tissue structure, the standard apparent diffusion coefficient *D*_app_, as thought to be correlated to increasing cell density, might be of higher diagnostic value since allowing to detect and quantify abnormal cell conglomerates [14].

Although *β*_FROC_ is thought to be related to structural complexity [23], a straightforward link to the underlying microstructure is difficult to unveil. One difficulty is that *β*_FROC_ values smaller than one, which were found in our study and in other works (e.g. [21–23]), correspond to an initial slope of infinite magnitude of the signal damping with respect to *b* at *b* = 0. As this initial signal damping at *b* = 0 is commonly thought to reflect the apparent diffusion coefficient averaged over the medium [34], it becomes difficult to relate the FROC parameter *D*_FROC_ to *D*_app_. One might argue that this initial slope is masked by the intravoxel incoherent motion (IVIM) effect anyway [29]. But by suppressing the blood signal [35] or by using flow-compensated diffusion encodings [36, 37], the IVIM effect can be suppressed so that this loophole is not available in general. Presumably one must restrict oneself to b-values larger than a certain threshold when using the FROC model. Maybe multi-compartment distribution models might be useful to gain insights on appropriate thresholds [38, 39]. Although not primarily being the scope of this manuscript, future in depth investigation of IVIM parameters as previously described to be helpful for lesion characterization might further contribute to DWI analyses of the breast with *D*_IVIM_ providing a high AUC.

There are certain limitations to this study. First, we did not obtain as many and high b-values as used in the previously described studies [21–23]. This might have influence on the calculation of the parameters and limit the results and makes it impossible to assess the correct functional form of the signal damping preventing a validation of the appropriateness of the model for the applications to the data of our study. This aspect needs to be considered, since the diagnostic performance of our FROC parameters thus might both be an over- or underestimation of the diagnostic potential of the method. A second limitation is found in the relatively low number of study subjects for benign and malignant lesions, which is, however, in the range of other studies investigating novel fitting methods in diffusion weighted imaging. Further the correlation between the X-ray mammography as the primary descriptor of the lesion and the MR mammography was done visually without invasive markers, thus miscorrelations cannot be completely ruled out, however are considered minor due to the double consensus reading for lesion definition by two radiologists. Since the described breast lesions are assumedly smaller than the investigated brain lesions. Further, signal to noise (SNR) varied substantial between the FROC derived maps, which can have a bias on the accuracy and precision of derived parameters. Another limitation is that it has been reported that the diagnostic accuracy of quantitative DWI analyses might be influenced by lesion size, and the here reported lesions were rather small compared to literature [40].

In conclusion, to our knowledge, this is the first description of using an advanced FROC fitting model for diffusion weighted imaging of the breast. DWI derived FROC parameters *β*_FROC_ and *μ*_FROC_ did not show a significant improvement in differentiating between malignant and benign breast lesions compared to apparent diffusion coefficient *D*_app_, which might be related to the structural heterogeneity of breast tissue or to the maximal b-value used.

## Supporting information

S1 Fig (EPS)

S2 Fig (EPS)

S1 Text On the coupling of *D*_FROC_ and *μ*_FROC_. (DOCX)

S2 Text Choice of b-values. (DOCX)

## References

[1] Smith R, Duffy S, Tabár L. Breast cancer screening: the evolving evidence. Oncology 2012 6(5):471–5, 479–81, 485–6. 22730603

[2] Lauby-Secretan B, Scoccianti C, Loomis D, Benbrahim-Tallaa L, Bouvard V, Bianchini F, et al Breast-Cancer Screening—Viewpoint of the IARC Working Group. New England Journal of Medicine. 2015;372(24):2353–8. doi: 10.1056/NEJMsr1504363 26039523 10.1056/NEJMsr1504363

[3] Bock K, Heywang-Kobrunner S, Regitz-Jedermann L, Hecht G, Kaab-Sanyal V. [Mammography screening in Germany. Current results and future challenges]. Der Radiologe. 2014;54(3):205–10. Epub 2014/03/14. doi: 10.1007/s00117-013-2581-7 24623009 10.1007/s00117-013-2581-7

[4] Alcusky M, Philpotts L, Bonafede M, Clarke J, Skoufalos A. The Patient Burden of Screening Mammography Recall. Journal of Women's Health. 2014;23(S1):S-11-S-9. 10.1089/jwh.2014.1511 25247382

[5] Berlin CMgKM. Evaluation-Report 2010. Results of the Mammography-Screening-Program in Germany. 2014.

[6] Kuhl CK. MR Imaging for Surveillance of Women at High Familial Risk for Breast Cancer. Magnetic Resonance Imaging Clinics of North America. 2006;14(3):391–402. doi: 10.1016/j.mric.2006.07.003 17098180 10.1016/j.mric.2006.07.003

[7] Kuhl CK, Schrading S, Strobel K, Schild HH, Hilgers R-D, Bieling HB. Abbreviated Breast Magnetic Resonance Imaging (MRI): First Postcontrast Subtracted Images and Maximum-Intensity Projection—A Novel Approach to Breast Cancer Screening With MRI. Journal of Clinical Oncology. 2014;32(22):2304–10. doi: 10.1200/JCO.2013.52.5386 24958821 10.1200/JCO.2013.52.5386

[8] Bickelhaupt S, Laun FB, Tesdorff J, Lederer W, Daniel H, Stieber A, et al Fast and Noninvasive Characterization of Suspicious Lesions Detected at Breast Cancer X-Ray Screening: Capability of Diffusion-weighted MR Imaging with MIPs. Radiology. 2015:150425. Epub 2015/09/30. 10.1148/radiol.2015150425 26418516

[9] Bickelhaupt S, Tesdorff J, Laun FB, Kuder TA, Lederer W, Teiner S, et al Independent value of image fusion in unenhanced breast MRI using diffusion-weighted and morphological T2-weighted images for lesion characterization in patients with recently detected BI-RADS 4/5 x-ray mammography findings. Eur Radiol. 2016. Epub 2016/05/20. 10.1007/s00330-016-4400-9 27193776

[10] Kul S, Oguz S, Eyuboglu I, Komurcuoglu O. Can unenhanced breast MRI be used to decrease negative biopsy rates? Diagnostic and interventional radiology (Ankara, Turkey). 2015;21(4):287–92. Epub 2015/04/04. PubMed Central PMCID: PMCPmc4498423. 10.5152/dir.2014.14333 PMC4498423 25835081

[11] Telegrafo M, Rella L, Stabile Ianora AA, Angelelli G, Moschetta M. Unenhanced breast MRI (STIR, T2-weighted TSE, DWIBS): An accurate and alternative strategy for detecting and differentiating breast lesions. Magn Reson Imaging. 2015;33(8):951–5. Epub 2015/06/29. doi: 10.1016/j.mri.2015.06.002 26117691 10.1016/j.mri.2015.06.002

[12] Radbruch A, Weberling LD, Kieslich PJ, Eidel O, Burth S, Kickingereder P, et al Gadolinium retention in the dentate nucleus and globus pallidus is dependent on the class of contrast agent. Radiology. 2015;275(3):783–91. Epub 2015/04/08. doi: 10.1148/radiol.2015150337 25848905 10.1148/radiol.2015150337

[13] Kanda T, Ishii K, Kawaguchi H, Kitajima K, Takenaka D. High signal intensity in the dentate nucleus and globus pallidus on unenhanced T1-weighted MR images: relationship with increasing cumulative dose of a gadolinium-based contrast material. Radiology. 2014;270(3):834–41. Epub 2014/01/31. doi: 10.1148/radiol.13131669 24475844 10.1148/radiol.13131669

[14] Thomassin-Naggara I, De Bazelaire C, Chopier J, Bazot M, Marsault C, Trop I. Diffusion-weighted MR imaging of the breast: Advantages and pitfalls. European Journal of Radiology. 2013;82(3):435–43. doi: 10.1016/j.ejrad.2012.03.002 22658868 10.1016/j.ejrad.2012.03.002

[15] Freitag MT, Bickelhaupt S, Ziener C, Meier-Hein K, Radtke JP, Mosebach J, et al [Selected clinically established and scientific techniques of diffusion-weighted MRI: In the context of imaging in oncology]. Der Radiologe. 2016;56(2):137–47. Epub 2016/01/24. doi: 10.1007/s00117-015-0066-6 26801187 10.1007/s00117-015-0066-6

[16] Wenkel E, Uder M, Janka R. Diffusion-weighted breast imaging. Der Radiologe. 2014;54(3):224–32. doi: 10.1007/s00117-013-2588-0 24570109 10.1007/s00117-013-2588-0

[17] Yabuuchi H, Matsuo Y, Sunami S, Kamitani T, Kawanami S, Setoguchi T, et al Detection of non-palpable breast cancer in asymptomatic women by using unenhanced diffusion-weighted and T2-weighted MR imaging: comparison with mammography and dynamic contrast-enhanced MR imaging. Eur Radiol. 2011;21(1):11–7. Epub 2010/07/20. doi: 10.1007/s00330-010-1890-8 20640898 10.1007/s00330-010-1890-8

[18] Baltzer PA, Benndorf M, Dietzel M, Gajda M, Camara O, Kaiser WA. Sensitivity and specificity of unenhanced MR mammography (DWI combined with T2-weighted TSE imaging, ueMRM) for the differentiation of mass lesions. Eur Radiol. 2010;20(5):1101–10. Epub 2009/11/26. doi: 10.1007/s00330-009-1654-5 19936758 10.1007/s00330-009-1654-5

[19] Partridge SC, Demartini WB, Kurland BF, Eby PR, White SW, Lehman CD. Differential diagnosis of mammographically and clinically occult breast lesions on diffusion-weighted MRI. J Magn Reson Imaging. 2010;31(3):562–70. Epub 2010/02/27. doi: 10.1002/jmri.22078 20187198 10.1002/jmri.22078

[20] Martelotto LG, Ng CKY, Piscuoglio S, Weigelt B, Reis-Filho JS. Breast cancer intra-tumor heterogeneity. Breast Cancer Research: BCR. 2014;16(3):210–. doi: 10.1186/bcr3658 25928070 10.1186/bcr3658 PMC4053234

[21] Sui Y, Wang H, Liu G, Damen FW, Wanamaker C, Li Y, et al Differentiation of Low- and High-Grade Pediatric Brain Tumors with High b-Value Diffusion-weighted MR Imaging and a Fractional Order Calculus Model. Radiology. 2015;277(2):489–96. doi: 10.1148/radiol.2015142156 26035586 10.1148/radiol.2015142156 PMC4627432

[22] Zhou XJ, Gao Q, Abdullah O, Magin RL. Studies of anomalous diffusion in the human brain using fractional order calculus. Magnetic resonance in medicine. 2010;63(3):562–9. Epub 2010/02/27. doi: 10.1002/mrm.22285 20187164 10.1002/mrm.22285

[23] Magin RL, Abdullah O, Baleanu D, Zhou XJ. Anomalous diffusion expressed through fractional order differential operators in the Bloch-Torrey equation. Journal of magnetic resonance (San Diego, Calif: 1997). 2008;190(2):255–70. Epub 2007/12/11. 10.1016/j.jmr.2007.11.007 18065249

[24] Sui Y, Xiong Y. Differentiation of Low- and High-Grade Gliomas Using High b-Value Diffusion Imaging with a Non-Gaussian Diffusion Model. AJNR American journal of neuroradiology. 2016;epub ahead of print. 10.3174/ajnr.A4836 PMC5018419 27256851

[25] Magin RL, Ingo C, Colon-Perez L, Triplett W, Mareci TH. Characterization of Anomalous Diffusion in Porous Biological Tissues Using Fractional Order Derivatives and Entropy. Microporous and mesoporous materials: the official journal of the International Zeolite Association. 2013;178:39–43. Epub 2013/09/28. PubMed Central PMCID: PMCPmc3780456. 24072979 10.1016/j.micromeso.2013.02.054 PMC3780456

[26] Bickelhaupt S, Laun F, Tesdorff J, Lederer W, Daniel H, Stieber A, et al Fast and non-invasive characterization of suspicious lesions detected on X-ray breast cancer screening–capability of diffusion weighted MRI with maximum intensity projections. Radiology. 2016;278(3):689–97. doi: 10.1148/radiol.2015150425 26418516 10.1148/radiol.2015150425

[27] Bickelhaupt S, Paech D, Kickingereder P, Steudle F, Lederer W, Daniel H, et al Prediction of malignancy by a radiomic signature from contrast agent-free diffusion MRI in suspicious breast lesions found on screening mammography. J Magn Reson Imaging. 2017. Epub 2017/02/06. 10.1002/jmri.25606 28152264

[28] Hering J, Tesdorff J, Laun FB, Lederer W, Daniel H, Stieber A, et al Applicability and discriminative value of a semi-automatic 3D spherical volume for the assessment of the apparent diffusion coefficient in unclear breast lesions using DWI–a feasibility study. Journal of Computed Assisted Tomography. 2015;in preparation.

[29] Le Bihan D, Breton E, Lallemand D, Aubin ML, Vignaud J, Laval-Jeantet M. Separation of diffusion and perfusion in intravoxel incoherent motion MR imaging. Radiology. 1988;168(2):497–505. Epub 1988/08/01. doi: 10.1148/radiology.168.2.3393671 3393671 10.1148/radiology.168.2.3393671

[30] Sun K, Chen X, Chai W, Fei X, Fu C, Yan X, et al Breast Cancer: Diffusion Kurtosis MR Imaging-Diagnostic Accuracy and Correlation with Clinical-Pathologic Factors. Radiology. 2015:141625. Epub 2015/05/06. 10.1148/radiol.15141625 25938679

[31] Wu D, Li G, Zhang J, Chang S, Hu J, Dai Y. Characterization of breast tumors using diffusion kurtosis imaging (DKI). PloS one. 2014;9(11):e113240 Epub 2014/11/19. PubMed Central PMCID: PMCPmc4236178. doi: 10.1371/journal.pone.0113240 25406010 10.1371/journal.pone.0113240 PMC4236178

[32] Iima M, Yano K, Kataoka M, Umehana M, Murata K, Kanao S, et al Quantitative non-Gaussian diffusion and intravoxel incoherent motion magnetic resonance imaging: differentiation of malignant and benign breast lesions. Invest Radiol. 2015;50(4):205–11. Epub 2014/09/27. doi: 10.1097/RLI.0000000000000094 25260092 10.1097/RLI.0000000000000094

[33] Koh D-M, Collins DJ, Orton MR. Intravoxel Incoherent Motion in Body Diffusion-Weighted MRI: Reality and Challenges. American Journal of Roentgenology. 2011;196(6):1351–61. doi: 10.2214/AJR.10.5515 21606299 10.2214/AJR.10.5515

[34] Jensen JH, Helpern JA. MRI quantification of non-Gaussian water diffusion by kurtosis analysis. NMR Biomed. 2010;23(7):698–710. Epub 2010/07/16. PubMed Central PMCID: PMCPmc2997680. doi: 10.1002/nbm.1518 20632416 10.1002/nbm.1518 PMC2997680

[35] Lemke A, Laun FB, Simon D, Stieltjes B, Schad LR. An in vivo verification of the intravoxel incoherent motion effect in diffusion-weighted imaging of the abdomen. Magnetic resonance in medicine. 2010;64(6):1580–5. Epub 2010/07/29. doi: 10.1002/mrm.22565 20665824 10.1002/mrm.22565

[36] Wetscherek A, Stieltjes B, Laun FB. Flow-compensated intravoxel incoherent motion diffusion imaging. Magnetic resonance in medicine. 2015;74(2):410–9. Epub 2014/08/15. doi: 10.1002/mrm.25410 25116325 10.1002/mrm.25410

[37] Ahlgren A, Knutsson L, Wirestam R, Nilsson M, Stahlberg F, Topgaard D, et al Quantification of microcirculatory parameters by joint analysis of flow-compensated and non-flow-compensated intravoxel incoherent motion (IVIM) data. NMR Biomed. 2016;29(5):640–9. Epub 2016/03/10. doi: 10.1002/nbm.3505 26952166 10.1002/nbm.3505 PMC5069652

[38] Pfeuffer J, Provencher SW, Gruetter R. Water diffusion in rat brain in vivo as detected at very large b values is multicompartmental. Magma (New York, NY). 1999;8(2):98–108. Epub 1999/08/24. 10.1007/BF02590526 10456372

[39] Yablonskiy DA, Bretthorst GL, Ackerman JJ. Statistical model for diffusion attenuated MR signal. Magnetic resonance in medicine. 2003;50(4):664–9. Epub 2003/10/03. PubMed Central PMCID: PMCPmc2140254. doi: 10.1002/mrm.10578 14523949 10.1002/mrm.10578 PMC2140254

[40] Wan CW, Lee CY, Lui CY, Fong CY, Lau KC. Apparent diffusion coefficient in differentiation between malignant and benign breast masses: does size matter? Clin Radiol. 2016;71(2):170–7. Epub 2015/12/22. doi: 10.1016/j.crad.2015.11.006 26688549 10.1016/j.crad.2015.11.006

