# Supplementary material for: On a fractional order calculus model in diffusion weighted breast imaging to differentiate between malignant and benign breast lesions detected on X-ray screening mammography
Source: PLoS One. 2017 Apr 28;12(4):e0176077. doi: 10.1371/journal.pone.0176077 (PMC5409173; doi:10.1371/journal.pone.0176077)
Supplement: S1 Text — (DOCX) [file pone.0176077.s003.docx]

**Supplemental Information 1:**

**S1 Text: On the coupling of** $\boldsymbol{D}_{\mathbf{FROC}}$ **and** $\boldsymbol{\mu}_{\mathbf{FROC}}$

The intention of the FROC approach is to determine the parameters $D_{\mathrm{FROC}}$, $\mu_{\mathrm{FROC}}$, and $\beta_{\mathrm{FROC}}$ by fitting Eq. 1 to the obtained signals, which are to be acquired with different $G$, and potentially with different $\delta$ and $\Delta$ [[21](#_ENREF_21), [22](#_ENREF_22)].

As a coupling of $D_{\mathrm{FROC}}$ and $\mu_{\mathrm{FROC}}$ appears to exist in Eq. 1, the question arises of whether both values can be determined unambiguously by use of Eq. 1.

This was tested numerically by computing $S$ for the parameter set $D_{1}=1 \mu m^{2}/ms$, $\beta_{1}=0.8$, and $\mu_{1}=8 \mu m$. The sequence parameters were chosen to be Δ=100 ms, δ=50 ms and 41 values of $G$ were used, which were equally distributed between 0 and 40 mT/m. All $D$-values are FROC parameters in S1. Two fits were performed with the Levenberg-Marquardt algorithm using as initial values once $D_{\mathrm{inital},1},=D_{1}$, $\beta_{\mathrm{inital},1}=\beta_{1}$, and $\mu_{\mathrm{inital},1}=\mu_{1}$ and once $D_{\mathrm{inital},2},=2D_{1}$, $\beta_{\mathrm{inital},2}=0.7$, and $\mu_{\mathrm{inital},2}=3\mu_{1}$.

With initial value set one, the fit yielded: $D_{\mathrm{fit}1}=1 \mu m^{2}/ms,$ $\beta_{\mathrm{fit}1}=0.8$, and $\mu_{\mathrm{fit}1}=8 \mu m$ with a root mean square error of 0. With initial value set two, the fit yielded: $D_{fit2}=1.552 \mu m^{2}/ms,$ $\beta_{fit2}=0.8$, and $\mu_{fit2}=24.02 \mu m$ with a root mean square error of 5.407e-08.

A second numerical test was performed using signals computed for $\delta=50 \mathrm{ms}$, and $\Delta=100 \mathrm{ms}$ and $\Delta=200 \mathrm{ms}$. The same values for $G$ were used. With initial value set one, the fit yielded: $D_{fit3}=1 \mu m^{2}/ms,$ $\beta_{fit3}=0.8$, and $\mu_{fit3}=8 \mu m$ with a root mean square error of 0. With initial value set two, the fit yielded $D_{fit4}=1.552 \mu m^{2}/ms,$ $\beta_{fit4}=0.8$, and $\mu_{fit4}=24.02 \mu m$ with a root mean square error of 4.815e-08.

A third numerical test was performed using signals computed for $\delta=25 \mathrm{ms}$ and $\delta=50 \mathrm{ms}$, and $\Delta=100 \mathrm{ms}$. The same values for $G$ were used. With initial value set one, the fit yielded: $D_{fit5}=1 \mu m^{2}/ms,$ $\beta_{fit5}=0.8$, and $\mu_{fit5}=8 \mu m$ with a root mean square error of 0. With initial value set two, the fit yielded $D_{fit6}=1.552 \mu m^{2}/ms,$ $\beta_{fit6}=0.8$, and $\mu_{fit6}=24.02 \mu m$ with a root mean square error of 6.665e-08.

Thus, apparently, none of the fits allow the unambiguous decoupling of $D_{\mathrm{FROC}}$ and $\mu_{\mathrm{FROC}}$, while $\beta_{\mathrm{FROC}}$ could be fitted reliably.

S1 Fig shows the computed signals (markers) and the fitted curves for the three tests, which were obtained with initial value set 2. The fitted curves and the signals are in perfect agreement although the fitted values of $D_{\mathrm{FROC}}$ and $\mu_{\mathrm{FROC}}$ deviate substantially from the true values. S1a Fig visualizes the first numerical test, S1b Fig the second numerical test, and S1c Fig the third numerical test.

Figuratively, the obtained numerical values can be interpreted as follows. The term $\left( \gamma G\delta\right)^{2\beta_{\mathrm{FROC}}}$ in Eq. 1 seems to fix the $\beta_{\mathrm{FROC}}$ parameter, so that $\beta_{\mathrm{FROC}}$ in the term $D_{\mathrm{FROC}}\mu_{\mathrm{FROC}}^{2\left( \beta_{\mathrm{FROC}}-1 \right)}$ can be regarded as a fixed constant. Consequently, it is only $D_{\mathrm{FROC}}\mu_{\mathrm{FROC}}^{2\left( \beta_{\mathrm{FROC}}-1 \right)}$ that can be obtained by fitting; for example, $D_{fit2}\mu_{fit2}^{2\left( \beta_{\mathrm{fit}2}-1 \right)}\approx D_{fit1}\mu_{fit1}^{2\left( \beta_{\mathrm{fit}1}-1 \right)}$.

**S1 Fig-:** **Coupling of** $\boldsymbol{D}_{\mathbf{FROC}}$ **and** $\boldsymbol{\mu}_{\mathbf{FROC}}$**.**

Markers represent computed signals. Curves represent fits to Eq. 1. Although the fitted values of $D_{\mathrm{FROC}}$ and $\mu_{\mathrm{FROC}}$ deviate substantially from the true values, the fitted curves match perfectly to the computed signals.
