# Supplementary material for: On a fractional order calculus model in diffusion weighted breast imaging to differentiate between malignant and benign breast lesions detected on X-ray screening mammography
Source: PLoS One. 2017 Apr 28;12(4):e0176077. doi: 10.1371/journal.pone.0176077 (PMC5409173; doi:10.1371/journal.pone.0176077)
Supplement: S2 Text — (DOCX) [file pone.0176077.s004.docx]

**Supplemental Information 2:**

**S 2: Choice of b-values**

The presented FROC evaluation was performed retrospectively using data from a study, which had not been designed prospectively for FROC imaging. For this reason, the used b-values had not been optimized for FROC model evaluations and deviate from earlier publications (such as [21]). The purpose of this Supplemental Information is to evaluate the appropriateness of the here used b-values 0, 100, 750, 1500 s/mm² (“b-value set 1”) in comparison to earlier used b-values 0, 10, 20, 50, 100, 200, 400, 800, 1200, 2000, 3000, 4000 s/mm² (“b-value set 2”, [21]).

For this purpose, computer simulations were performed assuming that the ground truth signal is given by the FROC model and either $D_{FROC,benign}$ = 1.35 µm²/ms, $\beta_{FROC,benign}$ = 0.75, and $\mu_{FROC,benign}$ = 8.91 µm; or $D_{FROC,malignant}$ = 1.03 µm²/ms, $\beta_{FROC,malignant}$ = 0.69, and $\mu_{FROC,malignant}$ = 8.34 µm. Gaussian noise was added 1000 times to the signal with a standard deviation of 0.01 for b-value set 1, which corresponds to an SNR of 100. To account for different number of b-values, the standard deviation of the noise was increased to 0.01$\cdot\sqrt{12/4}$ for b-value set 2. $D_{\mathrm{FROC}}$, $\beta_{\mathrm{FROC}}$, and $\mu_{\mathrm{FROC}}$ were fitted in the same manner as described in the manuscript^[[1]](#footnote-1)^. ( $D_{app, FROC}$ was initialized with a monoexponential fit using the b-values $\leq$ 1200 s/mm².)

S1 Table shows obtained mean values and standard deviations of the fitted values and S2 Fig shows boxplots of the obtained distributions.

The mean of $\beta_{\mathrm{FROC}}$ is obtained correctly with both of b-value sets, but the mean value of the fitted values for $D_{\mathrm{FROC}}$ and $\beta_{\mathrm{FROC}}$ deviate from the ground truth (owing to their coupling as described in Appendix 1). This deviation is larger for b-value set 2.

The standard deviations of fitted values are roughly reduced by 40 to 50% for b-value set 2. This evaluation does, however, not take into account the reduced signal that originates from longer echo times needed for the larger b-values of b-value set 2. The exact SNR reduction depends on available gradient amplitude and T2 time of the tissue. Roughly calculated, the necessary increase in diffusion time to reach a b-value of 4000 s/mm² instead of 1500 s/mm² is $\left( \frac{4000}{1500} \right)^{1/3}\approx1.387$. The diffusion time in our study was approximately $\Delta+\delta\approx50$ ms, thus the diffusion time needed for b=4000 s/mm² is roughly 70 ms. Assuming a transversal relaxation time of 50 ms, this decreases SNR to $\exp\left( -\frac{20}{50} \right)\approx0.67$, i.e. by 33%. Taking this factor into account, it can be stated that b-value set 1, which was used in our study, allows one to estimate the FROC parameters with a reasonable precision in comparison to b-value set 2.

S1 Table: Fitted FROC parameters in computer simulations evaluating the appropriateness of the b-value distribution used in this study (“b-value set 1”) in comparison to the “b-value set 2” used in [21].

| b-value set |  | $D_{\mathrm{FROC}}$ [µm²/ms] | $\beta_{\mathrm{FROC}}$ | $\mu_{\mathrm{FROC}}$ [µm] |
| --- | --- | --- | --- | --- |
| ground truth | malignant | 1.03 | 0.69 | 8.34 |
| 1 |  | 0.908 ± 0.048 | 0.690 ± 0.055 | 6.80 ± 0.29 |
| 2 |  | 0.831 ± 0.031 | 0.690 ± 0.029 | 5.89 ± 0.16 |
| ground truth | benign | 1.35 | 0.75 | 8.91 |
| 1 |  | 1.200 ± 0.060 | 0.754 ± 0.051 | 7.04 ± 0.28 |
| 2 |  | 1.145 ± 0.039 | 0.750 ± 0.031 | 6.40 ± 0.15 |

**S2 Fig: Fitted FROC parameters in computer simulations.**

Fitted FROC parameters in computer simulations evaluating the appropriateness of the b-value distribution used in this study (“b-value set 1”) in comparison to the “b-value set 2” used in [21] for “benign” and “malignant” FROC parameters. Note that the SNR decrease owing to prolonged echo times for b-value set 2 is not taken into account in this plot (see text).

1. [↑](#footnote-ref-1)
